# Is shared decision-making a determinant of polypharmacy in older patients with chronic disease? A cross-sectional study in Hubei Province, China

**DOI:** 10.1186/s12877-023-03968-1

**Published:** 2023-04-29

**Authors:** Qiao Zong, Zhanchun Feng, Jia Wang, Zehao Zheng, Chaoyi Chen, Da Feng

**Affiliations:** 1 School of Medicine and Health Management, Tongji Medical College, Huazhong University of Science and Technology, Wuhan, People’s Republic of China; 2 School of Pharmacy, Tongji Medical College, Huazhong University of Science and Technology, Wuhan, People’s Republic of China

**Keywords:** Shared decision-making, Polypharmacy, Chronic disease, Informed decision-making, Paternalistic decision-making

## Abstract

**Background:**

Shared decision-making(SDM) is recognized as an important means of managing polypharmacy among older people with chronic diseases. However, no studies have quantitatively measured the effect of SDM on polypharmacy. The objective of this study was to compare the impact of SDM and other factors on polypharmacy in inpatients and community patients. Additionally, the study aimed to compare the impact of different decision types on polypharmacy in community patients.

**Methods:**

This is a population-based multicenter retrospective study conducted in Hubei Province, China. A cluster sampling approach was used to recruit 536 chronic disease inpatients from March to April 2019, and 849 community patients were recruited from April to June 2021. Propensity score weighting was used to control the confounding variables and determine the net effect of SDM on polypharmacy.

**Results:**

Among the 536 hospitalized patients, the prevalence of polypharmacy was 56.3%. A high level of SDM was significantly associated with a lower risk of polypharmacy. Patients with chronic illnesses aged 76 years and older and with an annual family income of 24,001–36,000 yuan were associated with a lower likelihood of polypharmacy (*p* < 0.05). Multimorbidity was often accompanied by the occurrence of multiple medication use. Among 849 community patients, the prevalence of polypharmacy was 21.8%. Among types of decision-making, informed and paternalistic decision-making showed a higher likelihood of polypharmacy compared with shared decision-making (*P* < 0.05). Male, older patients over 76 years of age, urban residents, annual household income of 12,001–24,000 yuan, and multimorbidity were associated with higher likelihood of polypharmacy (*P* < 0.05). Patients with an annual household income of 24,001–36,000 yuan, 36,001 yuan or more, and good medication compliance showed a lower likelihood of polypharmacy (*P* < 0.05).

**Conclusions:**

The prevalence of polypharmacy is high among China's older population with chronic disease who should be paid more atthention by the healthcare providers. Additionaly, encouraging the patients' attendance in SDM, reducing paternalistic and informed decision-making during prescribing, improving patient medication compliance, and increasing the promotion and guidance of rational medication use for patients are essential to reduce polypharmacy in Chinese chronic disease patients.

## Introduction

Under the influence of increasing life expectancy and aging in China, chronic non-communicable diseases have gradually being a major disease burden in China [1]. Polypharmacy is a common condition and challenge in managing patients with chronic diseases [2]. It occurs when patients regularly take 5 or more medications [3]. According to a research, the incidence of polypharmacy in patients with chronic kidney disease in Europe is as high as 91% [4]. A cross-sectional study in China has found that more than 75% of diabetes mellitus type 2(T2DM) patients were prescribed polypharmacy [5]. In some cases, polypharmacy is clinically considered appropriate but inappropriate most of the time [6]. Drug-drug interactions and drug-disease interactions caused by polypharmacy have been shown to be the key factors leading to a variety of adverse outcomes, such as mortality, adverse drug reactions, dysfunction, and prolonged hospital stays [7]. Therefore, how to effectively govern polypharmacy has become an urgent public health issue [8].

Experts are actively looking for ways to manage polypharmacy and its safety risks for patients. Shared decision-making (SDM) is a model for contemporary clinical decision-making, identified by the American Institute of Medicine as the key way incorporated into the UK's National Health System to improve healthcare quality [9, 10]. Specifically, SDM refers to the process by which medical staff and patients discuss the strengths and weaknesses of various therapeutic regimens, consider the patient's values and preferences, and encourage patients to participate in decision-making [11]. The traditional clinical decision-making model includes three types: paternalistic decision-making, informed decision-making, and SDM [12]. Among them, paternalistic decision-making is one of the important reasons for polypharmacy [13]. It is because the clinicians were always lacking of communication with patients before making clinical decisions and cloud not fully take the patients' perceptions for treatment goals, medications, and chronic diseases into account[14]. Some studies have shown that SDM is associated with increased patients’ engagement, satisfaction, compliance, and reduced hospitalization rates [15]. Moreover, many studies have confirmed the role of SDM in the management of various clinical patients [16–18]. These studies suggest that SDM can assist doctors and patients in selecting a more effective treatment plan. Experts have reached a consensus that SDM is an essential tool for managing polypharmacy in patients with chronic illnesses [19]. However, there was no quantitative measurement to calculate the impact of SDM on polypharmacy.

To provide evidence that the net impact of SDM on polypharmacy, this study conducted a cross-sectional survey based on inpatients and community patients in Hubei Province, China. Based on the requirements of China's "Basic Public Health Services", patients with hypertension and diabetes are the main groups the primary care providers focus on [20]. The patients have higher medication needs and potentially higher risk of polypharmacy. Therefore, we will focus on diabetes and hypertension in our community-based survey. The purpose of this study was to compare the effects of SDM and other factors on polypharmacy in inpatients and community patients. Also, to compare the impact of different decision-making types on polypharmacy in community patients. It aims to fill the gap in the relevant research field and provide an empirical basis for the subsequent formulation of interventions to reduce polypharmacy.

## Methods

### Study design and study site

This cross-sectional study was conducted by the cluster sampling method from March to April 2019 for inpatients and from April to June 2021 for community patients in Hubei Province, China.

### Study population

To collect data on inpatients, we first divided the 13 administrative districts in Wuhan City, Hubei Province, China into lots, stirred them well, and then selected 8 administrative districts by simple random sampling. We then randomly selected 8 hospitals from the tertiary hospitals located in these administrative districts in the same sampling method. Patients aged 66 years or older with at least one chronic disease (e.g., hypertension, heart disease, and diabetes) who had been on daily medication for 3 months or more were recruited to participate in the survey. We ultimately collected 603 questionnaires from inpatients, of which 536 were valid, with a valid response rate of 88.9%.

### Inclusion and exclusion cretieria

When collecting data on community patients. We first distinguished between urban and rural areas in Hubei Province, and then we followed the same simple randomization process described above and sampled 2 cities in each of them(in total of 4). We randomly sampled 3 towns in each of the four cities, resulting in a total of 12 towns. And each town needs to collect 100 patients diagnosed with hypertension or diabetes, which can be managed by primary health care providers. Community patients’ inclusion criteria: 1) Patient age > 65 years old; 2) Patients who meet the clinical diagnostic criteria for chronic diseases; 3) Patients who have taken medicines for a long time period of three months or more due to chronic diseases; 4) Patients with clear consciousness and can correctly express their wishes; 5) Patients without mental and psychological diseases. Exclusion criteria: 1) Patients with acute complications; 2) People who have not taken the medications for more than 3 months. A total of 858 participants participated in the survey, 9 of whom were excluded due to lack of information, of which 849 were valid. Effective response rate was 98.9%.

### Data collection tool

The paper version of the anonymous standardized structure questionnaire was used to collect data for this study. Prior to the start of the survey, each patient is required to complete an informed consent form or give verbal consent to participate in the survey. The entries in the questionnaire include socio-demographic characteristics, i.e., gender, age, domicile, educational background, and family annual income. Disease-related conditions include the number of chronic diseases and medication compliance. Because we assumed a high medication compliance for inpatients, we investigated this variable in community patients.

### Measurement

Polypharmacy is defined as regularly taking 5 or more medications [3]. Nutraceuticals are not included. In hospitalized patients, the number of medications which refers to the patient takes for the treatment in hospital.

We used the SDM-9 questionnaire to assess the level of SDM [21]. Entries range in ratings from "0" to "5" with a total score of 0–45 per participant, with a score conversion range of 0–100 multiplied by 20/9. The level of SDM for the respondent and the doctor was evaluated by using the mean of the conversion scale score as the bounding value. The overall Cronbach’$\alpha$ coefficient of the scale was 0.98.

We used the Control Preference Scale-Post (CPSpost) to assess the type of decision-making in community patients [22, 23], that is, the actual control of doctors and patients over medical decisions. The types of decision-making were divided into SDM, informed decision-making, and paternalistic decision-making.

### Data analysis

A descriptive analysis of sample characteristics was performed on the Statistical Analysis System (SAS) version 9.4 for Windows (SAS Institute Inc., Cary, NC, USA), and the other statistical analyses were performed on R Commander Version 4.04. The Pearson’s *χ2* test was used to conduct descriptive analyses of sociodemographic characteristics and disease-related factors in different polypharmacy status groups, as well as the differences between the underlying conditions of inpatients and community patients. In addition, the correlation between SDM and polypharmacy was explored by PSW (propensity-score weighting), and the OR (odds ratio) and 95% CI (confidence interval) of the variables were reported. Finally, a marginal effect analysis was performed for each variable. All analyses were conducted at the 0.05 level of statistical significance.

## Results

1. Characteristics of participants and single-factor analysis

This study surveyed older people with chronic diseases who are prone to accessing polypharmacy in Hubei Province, China. Table 1 shows the differences in demographic characteristics between the groups defined by polypharamcy among inpatients and community patients.

**Table 1:** Characteristics of participants and prevalence of polypharmacy among inpatients and community patients

Variables	Inpatients							Community patients						
	***n***	**%**	**polypharmacy**				***P*-value**	***n***	**%**	**Polypharmacy**				***P*-value**
			**Yes**		**No**					**Yes**		**No**		
			***n***	**%**	***n***	**%**				***n***	**%**	***n***	**%**	
**Total**	536	100	302	56.3	234	43.7		849	100.0	185	21.8	664	78.2	
**Gender**							0.089							0.856
male	212	39.5	129	42.7	83	35.47		353	41.6	78	42.2	275	41.4	
female	324	60.5	173	57.3	151	64.53		496	58.4	107	57.8	389	58.6	
**Age**							0.382							0.022
66–75	284	53.0	155	51.3	129	55.1		740	87.2	152	82.2	588	88.6	
≥ 76	252	47.0	147	48.7	105	44.9		109	12.8	33	17.8	76	11.4	
**Domicile**							0.088							0.069
Urban	402	25.0	235	77.8	167	28.6		492	58.0	118	63.8	374	56.3	
Rural	134	75.0	67	22.2	67	71.4		357	42.0	67	36.2	290	43.7	
**Education**							0.750							0.229
Primary school and below	183	34.1	104	34.4	79	33.8		407	47.9	83	44.9	324	48.8	
Junior high school	163	30.4	87	28.8	76	32.5		246	29.0	56	30.3	190	28.6	
Senior high school and above	190	35.5	111	36.75	79	33.7		196	23.1	46	24.9	150	22.6	
**Family annual income/yuan**							0.135							0.354
≤ 12,000	60	11.2	37	12.2	23	9.8		257	30.3	48	26.0	209	31.5	
12,0001–24,000	45	8.4	23	7.6	22	9.4		60	7.1	20	10.8	40	6.0	
24,001–36,000	95	17.7	31	10.3	64	27.4		95	11.2	16	8.7	79	11.9	
≥ 36,001	336	62.7	211	69.9	125	53.4		437	51.5	101	54.6	336	50.6	
**Number of chronic diseases**							< 0.001							< 0.001
1 disease	79	14.7	27	8.9	52	22.2		333	39.2	19	10.3	314	47.3	
2 diseases	150	28.0	72	23.8	78	33.3		271	31.9	40	21.6	231	34.8	
3 diseases and above	307	57.3	203	67.2	104	44.4		245	28.9	126	68.1	119	17.9	
**Compliance**							-							0.004
Yes	-	-	-	-	-	-		530	62.4	99	53.5	431	64.9	
No	-	-	-	-	-	-		319	37.6	86	46.5	233	35.1	
**SDM**							0.943							0.996
Low	289	53.9	176	58.3	113	48.3		560	66.0	122	65.9	438	66.0	
High	247	46.1	126	41.7	121	51.7		289	34.0	63	34.1	226	34.0	
**Type of Decision-Making**							-							0.457
Informed	-	-	-	-	-	-		297	35.0	63	34.1	234	35.2	
SDM	-	-	-	-	-	-		45	5.3	8	4.3	37	5.6	
Paternalistic	-	-	-	-	-	-		507	59.7	114	61.6	393	59.2	

*SDM* Shared decision making

Among inpatients, up to 56.3% reported polypharmacy. Less than half of the patients reported high levels of SDM. 60.5% of inpatients were female. More than half of patients were between 66 and 75 years old. Long-term urban residents accounted for 3/4 of the patients. 57.3% of the hospitalized patients had 3 or more chronic diseases.

Among the community patients, 21.8% of patients reported polypharmacy. Only 34.0% patients had high levels SDM. The vast majority of patients reported a predominance of paternalistic decision making when receiving medical care (59.7%), followed by informed decision making (35.0%). SDM was reported by only 5.3% of patients. The majority of participants were female (58.4%). The vast majority of patients were distributed between 66 and 75 years of age (87.2%), with a predominantly urban population (58.0%). Nearly half of community patients had only primary school education or less (47.9%), and more than half of patients had an annual family income of more than 36,000yuan. In addition, 28.9% of patients had 3 or more diseases and 62.4% had good medication compliance.

2. Results of propensity score weighting(PSW)

The standardized mean difference was used to assess the balance of variables between the experimental and control groups before and after weighting, and a threshold value of 0.20 was set. If the weighted standardized mean difference was greater than 0.2, there was no balance. As shown in Table 2, two variables, level of SDM and type of decision-making, were added to the regression equations, respectively. Controlling for other variables that remained unchanged. After the propensity score weighting (PSW), the covariates with large deviations in the original data were balanced and the overall distribution of the data was much more balanced than before.

**Table 2:** The standardized mean difference of each covariable before and after weighting

Variable	Standardized mean difference					
	Inpatients		Community patients			
	Level of SDM		Level of SDM		Type of decision-making	
	Before weight	After weight	Before weight	After weight	Before weight	After weight
Gender	0.15	-0.04	0.02	-0.01	0.29	0.00
Age	0.08	-0.03	0.18	-0.02	0.12	0.14
Domicile	0.15	0.03	0.15	0.02	0.28	0.06
Education	0.03	0.05	0.10	-0.04	0.19	0.15
Family annual income	0.13	0.00	0.07	-0.05	0.12	0.11
Number of chronic diseases	0.50	0.00	1.22	0.00	0.31	0.06
Compliance	-	-	-0.23	-0.05	0.55	0.09

Table 3 shows the results of PSW in inpatients. It is noteworthy that a high level of SDM were less likely to have polypharmacy (OR = 0.64, 95% CI: 0.44–0.94). In addition, older patients over 75 years of age (OR = 1.57, 95% CI: 1.06–2.34), with 2 diseases (OR = 1.87, 95% CI = 1.04–3.42) and 3 or more diseases (OR = 4.57, 95% CI = 2.63–8.11) were more likely to have polypharmacy.

**Table 3:** Multivatiable logistic regression of factors associated with polypharmacy in inpatients after PSW and effect of SDM on polypharmacy

	**OR(95%CI)**	**Marginal Effects** **(95% CI)**	***P*-value**
**SDM (ref = Low)**			
High	0.64 (0.44–0.94)	-0.09 (-0.17- -0.01)	0.022
**Gender (ref = Female)**			
Male	1.44 (0.97–2.13)	0.08 (0.00–0.16)	0.072
**Age (ref = 66–75)**			
≥76	1.57 (1.06–2.34)	0.09 (0.01–0.18)	0.026
**Domicile (ref = Rural)**			
Urban	1.35 (0.86–2.12)	0.06 (-0.03- 0.16)	0.189
**Education (ref = Primary School and Below)**			
Junior high school	0.95 (0.59–1.54)	-0.01 (-0.11- 0.09)	0.841
Senior high school and above	0.77 (0.48–1.23)	-0.05 (-0.15- 0.04)	0.282
**Family annual income/yuan (ref = ~ 12,000)**			
12,0001–24,000	0.57 (0.25–1.30)	-0.12 (-0.30- 0.06)	0.181
24,001–36,000	0.22 (0.10–0.45)	-0.33 (-0.48- -0.18)	< 0.001
36,001 ~	0.89 (0.47–1.66)	-0.02 (-0.15- 0.11)	0.727
**Number of chronic disease (ref = 1 disease)**			
2 diseases	1.87 (1.04–3.42)	0.14 (0.01- 0.26)	0.039
3 diseases and above	4.57 (2.63–8.11)	0.33 (0.22–0.45)	< 0.001

*SDM* Shared decision making

Tables 4 and 5 demonstrate the results of the logistic regression after PSW among community patients. The results in Table 4 show that the level of SDM among community patients was not significantly associated with the occurrence of polypharmacy. Patients from urban areas (OR = 2.28, 95%CI = 1.29–4.11), patients with an annual family income of 12,001–24,000 yuan (OR = 2.34, 95%CI = 1.10–4.90), patients with 2 diseases (OR = 3.09, 95%CI = 1.75–5.64) and 3 or more diseases (OR = 22.40, 95%CI = 13.07–40.34) were more likely to develop polypharmacy.

**Table 4:** Multivatiable logistic regression of factors associated with polypharmacy in community patients after PSW and effect of SDM on polypharmacy

Variables	**OR (95%CI)**	**Marginal Effects (95% CI)**	***P*-value**
**SDM (ref = Low)**			
High	0.92 (0.61–1.38)	-0.01 (-0.06–0.04)	0.722
**Gender (ref = Female)**			
Male	1.26 (0.84–1.89)	0.03 (-0.03–0.08)	0.264
**Age (ref = 66–75)**			
≥76	1.55 (0.89–2.66)	0.06 (-0.02–0.13)	0.115
**Domicile (ref = Rural)**			
Urban	2.28 (1.29–4.11)	0.10 (0.03–0.17)	0.005
**Education (ref = Primary School and Below)**			
Junior high school	1.14 (0.68–1.91)	0.02 (-0.05–0.08)	0.610
Senior high school and above	1.29 (0.70–2.39)	0.03 (-0.05–0.11)	0.415
**Family annual income/yuan (ref = ~ 12,000)**			
12,0001–24,000	2.34 (1.10–4.90)	0.12 (0.01–0.24)	0.025
24,001–36,000	0.54 (0.25–1.15)	-0.07 (-0.15–0.02)	0.117
36,001 ~	0.88 (0.49–1.60)	-0.02 (-0.09–0.06)	0.679
**Compliance (ref = No)**			
Yes	0.79 (0.54–1.17)	-0.03 (-0.08–0.02)	0.242
**Number of chronic disease (ref = 1 disease)**			
2 diseases	3.09 (1.75–5.64)	0.09 (0.05–0.14)	< 0.001
3 diseases and above	22.40 (13.07–40.34)	0.48 (0.41–0.54)	< 0.001

*SDM* Shared decision making

**Table 5:** Multivatiable logistic regression of factors associated with polypharmacy in community patients after PSW and effect of type of decision-making on polypharmacy

Variables	**OR (95%CI)**	**Marginal Effects (95% CI)**	***P*-value**
**Type of Decision-Making (ref = **SDM**)**			
Informed	2.14 (1.46–3.17)	0.08 (0.04–0.12)	< 0.001
Paternalistic	2.43 (1.66–3.59)	0.09 (0.06–0.13)	< 0.001
**Gender (ref = Female)**			
Male	1.37 (1.03–1.83)	0.04 (0.00–0.07)	0.028
**Age (ref = 66–75)**			
≥76	1.59 (1.07–2.35)	0.06 (0.00–0.11)	0.020
**Domicile (ref = Rural)**			
Urban	2.94 (1.97–4.44)	0.12 (0.08–0.17)	< 0.001
**Education (ref = Primary School and Below)**			
Junior high school	1.17 (0.82–1.66)	0.02 (-0.02–0.06)	0.385
Senior high school and above	1.30 (0.85–2.01)	0.03 (-0.02–0.08)	0.228
**Family annual income/yuan (ref = ~ 12,000)**			
12,0001–24,000	2.08 (1.20–3.56)	0.10 (0.02–0.18)	0.008
24,001–36,000	0.42 (0.24–0.71)	-0.10 (-0.15- -0.04)	0.001
36,001 ~	0.66 (0.45–0.99)	-0.05 (-0.10- 0.00)	0.044
**Compliance (ref = No)**			
Yes	0.57 (0.44–0.74)	-0.07 (-0.10- -0.04)	< 0.001
**Number of chronic disease (ref = 1 disease)**			
2 diseases	3.17 (2.13–4.79)	0.09 (0.06–0.12)	< 0.001
3 diseases and above	27.46 (18.73–41.23)	0.49 (0.44–0.53)	< 0.001

*SDM* Shared decision making

The results in Table 5 show that among community patients, informed decision-making (OR = 2.14, 95% CI = 1.46–3.17) and paternalistic decision-making (OR = 2.43, 95% CI = 1.66–3.59) were associated with higher risk of polypharmacy compared to SDM. In addition, male patients (OR = 1.37, 95% CI = 1.03–1.83), older patients aged 76 years or older (OR = , 95% CI =), patients in urban areas (OR = 2.94, 95% CI = 1.97–4.44), patients with annual family income of 12,000–24,000 yuan (OR = 2.08, 95% CI = 1.20- 3.56), and patients with 2 diseases (OR = 3.17, 95% CI = 2.13–4.79) and 3 or more diseases (OR = 27.46, 95% CI = 18.73–41.23) were more likely to develop polypharmacy. Patients with a family income of 24,001–36,000 yuan(OR = 0.42, 95% CI = 0.24–0.71) and 360,000 yuan or more (OR = 0.66, 95% CI = 0.45–0.99), and high medication compliance (OR = 0.57, 95% CI = 0.44–0.74) were less likely to have polypharmacy.

## Discussion

We counted the prevalence of polypharmacy in older people hospitalized patients and community patients in China. Also, we analyzed the relationship between the level of SDM and different types of decision-making and polypharmacy. We found a prominent prevalence of polypharmacy in China, with about 56.3% of inpatients and 21.8% of community patients receiving polypharmacy, respectively. Besides, higher SDM level was significantly associated with lower risk of polypharmacy (OR = 0.64, 95% CI: 0.44–0.94). In the community, informed decision-making(OR = 2.14, 95% CI = 1.46–3.17) and paternalistic decision-making (OR = 2.43, 95% CI = 1.66–3.59) are more likely to occur with polypharmacy compared with SDM.

The prevalence of polypharmacy in community patients reported in this study (21.8%) was lower than that in other studies. A national cohort study conducted by Tae et al. in South Korea reported a 46.6% prevalence of polypharmacy [24]. In a community-based cohort study conducted by Shahar et al. in the southern United States, 57% of residents reported polypharmacy [25]. The prevalence reported in this study is much lower than those of other studies, which may be related to the different composition of the types of participants in the sample. 51.7% of patients in our study were from rural areas, compared to only 13.7% of rural patients in Tae's study [24]. The higher risk of polypharmacy among urban residents was also confirmed in the PSW results of community patients. The reason might be that compared with patients living in urban areas for a long time, the economic level of rural patients is lower, and the proportion of affordable drugs to rural residents is lower [26]. Polypharmacy will increase the cost of medicine for patients. In addition, easier access to medicine in urban areas is also a possible reason. For urban dwellers, the convenience of pharmacies increases their chances of developing self-medication behaviors.

At the same time, we speculate that the reason why the prevalence of polypharmacy in community patients (21.8%) is lower than that of hospitalized patients (53.6%) might be related to poor drug adherence in patients. 37.6% of community patients reduced medications during treatment. Studies have shown that forgetfulness due to aging, lack of understanding, physical problems and overly complex treatment regimens might cause patients to inadvertently disobey medical advices. [27, 28]. This can be avoided in inpatients with the help of nursing staff. Secondly, it might also because patients take self-medication based on empiricism, freely choosing the type and number of medications to take [29]. Compared to community patients, inpatients may have a greater need for medication to manage their condition. Moreover, they are not likely to reduce their medications arbitrarily under the supervision of healthcare professionals. Therefore, inpatients are more likely to have polypharmacy.

In our results, the prevalence of a high level of SDM was 46.1% and 34.0% in inpatients and community patients, respectively. SDM was more common among inpatients, which might be related to the higher opportunities for physician–patient communication during hospitalization. For example, physicians need to know all aspects of the patient's condition in-depth during hospitalization to complete the medical record, and they need to be constantly aware of the patient's condition and feelings during physician rounding [30]. The proximity of the patient's room to the physician's office makes it easier to approach the physician to ask questions about the treatment plan. These create a better atmosphere for SDM. Community patients lack this advantage.

We found that inpatients with a higher SDM had a lower prevalence of polypharmacy compared with patients with a lower level of SDM. A review of Peron et al. of the management of T2DM patients reveals that SDM can help provide more goal-oriented care [31]. Modern medicine is shifting from the traditional paternalistic decision-making model to a SDM model in which expertise flows in both directions. For medical staff, they can not only rationally choose evidence-based information but also reduce the occurrence of repeated prescriptions [9, 32]. Patients will have a clearer understanding of their medication regimen, so that they can give timely feedback on their needs to the doctors, whether they what to give up or continue treatment [31]. This might allow doctors to reduce unnecessary medication prescriptions. In community patients, this study did not find a relationship between SDM and polypharmacy, which might be due to the fact that most community patients are in a state of good disease control and the probability of polypharmacy is low.

This study found that among the three major decision-making types, both informed decision making and paternalistic decision making were associated with a higher risk of polypharmacy among patients in the community compared to SDM. The results of marginal effects analysis also confirmed that these two decision-making types explained 8% (95% CI: 0.04–0.14) and 9% (95% CI: 0.06–0.13) of the risk of polypharmacy, respectively. We suggest that one possible explanation for the greater risk of polypharmacy with informed decision-making is that patients do not have an adequate background knowledge base in medicine. Informed decision-making is more often based on the patient’s empiricism. This might lead to incidents such as repeated prescriptions and unreasonable self-medication. Paternalistic decision-making was the most dominant form of decision-making in the past, where the physician was seen as an authority figure and dominated the choice of treatment options while the patient cooperated with the treatment [13]. However, in the last 20 years there has been a general consensus worldwide that SDM should gradually replace paternalistic decision-making [33]. This is because in the context of paternalistic decision-making, the core of the physician's work is to treat the current disease. However, in the actual visit, due to the increasing complexity of health care, physicians need to meet more of the patient's needs, which includes diagnostic uncertainty prior to effective treatment planning, and to monitor or stop previously inappropriate medications. Rather than standardizing treatment for a disease, physicians must understand the patient's perception of the disease and treatment expectations in order to develop a personalized plan of care that addresses the patient's critical issues. In contrast to informed and paternalistic decision-making, SDM provides the physician with the background knowledge and understanding of the patient's existing medication regimen, while also taking into account the patient's own needs. Therefore, SDM can help both physicians and patients make mutually satisfactory treatment plans that strike a balance between aggressive treatment of disease and avoidance of causing drug-related harm in polypharmacy [34].

There is also a relationship between gender and the occurrence of polypharmacy. This study shows that men in wards have a higher risk of polypharmacy. A cross-sectional study in Europe showed that among patients with chronic diseases over the age of 80, men were more likely to have polypharmacy [35]. On the one hand, this might be related to the higher number of chronic diseases in older people. On the other hand, when symptoms of the disease appear, men often choose to ignore them. And when they have to be treated, the condition often needs to be controlled with more drugs [36].

In addition, our study showed that an annual family income of 12,001–24,000 yuan (in the low to moderate income group in this study) was associated with a higher risk of polypharmacy. In contrast, patients with annual family incomes of 24,001–36,000 yuan (middle to high income group) and 36,000 yuan or more (high income groups) were less likely to experience SDM than those in the low-income group (0–12,000 yuan). The latter result is consistent with the conclusions of a cohort study in Denmark [37]. Conversely, a study has also reported a correlation between high-income chronic disease patient groups and a higher prevalence of SDM [38]. This discrepancy between the findings might stem from differences in educational attainment, health literacy, and other characteristics of different income groups. Therefore, further research is needed to explore the relationship between income and SDM.

In community patients, the higher the adherence, the lower the likelihood of multiple medication use occurring. In previous studies, several studies have confirmed the relationship between multiple medication use and adherence, and similar conclusions were obtained to our study [28]. Previous studies have suggested that an increase in the number of prescribed medications and complexity of treatment regimens can exacerbate patients' difficulties in taking their medications, resulting in lower medication adherence [39]. As the present study was only cross-sectional, we could only confirm the association between adherence and multiple medication use, but not further the sequence between the two.

The occurrence of polypharmacy is often co-existent with multimorbidity [6]. This has been recognized by the academic community, and our research once again provides proof of this idea. It is worth mentioning that our study shows that polypharmacy shows an exponential growth trend as the disease increases. The results of the marginal effects showed that when the number of diseases increased from 1 to 3 or more, the marginal effect increased by 37% (the probability of polypharmacy increased by 37%) in inpatients and increased by 0.44 (the probability of polypharmacy increased by 44%) in community patients.

### Limitations

Our study still has certain limitations. Firstly, there is a limitation with the definition. When we defined polypharmacy, this study only considered quantity (≥ 5) and did not consider the possible rational use of it. However, our research still reflects the fact that the prevalence of polypharmacy in patients with chronic diseases in China is still high.

The next limitation is representativeness and accuracy. As the survey was conducted in only one province, its findings may not be applicable to other regions of China. To overcome the limitations of the study, future studies could be conducted in various regions of China to better understand the prevalence of polypharmacy and the impact of SDM nationwide. Additionally, the results of this study were based on patients' self-reported medication use, which may be influenced by recall bias. Objective measures, such as pharmacy records, could be used in the future to track medication use and reduce the effect of recall bias on the results.

Finally, there are limitations in the study design section. Although we attempted to explore the role of SDM in polypharmacy in different populations by comparing the differences between inpatients and community patients, the principles cannot be further clarified due to the insufficient variables included in the study. However, our results suggest that SDM can have different effects in different populations. In addition, this study did not examine the quality of SDM or the specific techniques used, which may have influenced the results. To address this issue, future studies could use validated measures to assess the quality of SDM and evaluate how different SDM techniques affect polypharmacy. Furthermore, this study did not investigate the potential adverse effects of polypharmacy, which should be refined in future studies to help understand the risks associated with the consumption of polypharmacy.

## Conclusions

In short, the prevalence of polypharmacy in older patients with chronic diseases remains high in China, especially among inpatients. In the future management of patients with chronic diseases, we suggest that healthcare providers pay more attention to key populations, encourage patients to participate in SDM at the time of consultation, and reduce paternalistic and informed decision-making in the prescription process to seek the best treatment options for chronic disease management. In addition, measures should be taken to improve patients' medication compliance and increase the promotion and guidance of rational medication use for patients in urban areas. The above measures are essential to reduce polypharmacy in Chinese patients with chronic diseases.

## Data Availability

The datasets analysed in the current study are not publicly available due to the fact that the data involves patients’ personal information but are available from the corresponding author on reasonable request.
